# An Effective Method to Measure Disease Similarity Using Gene and Phenotype Associations

**DOI:** 10.3389/fgene.2019.00466

**Published:** 2019-05-21

**Authors:** Shuhui Su, Lei Zhang, Jian Liu

**Affiliations:** ^1^School of Computer Science and Technology, Harbin Institute of Technology, Harbin, China; ^2^School of Biological and Chemical Engineering, Zhejiang University of Science and Technology, Hangzhou, China

**Keywords:** disease similarity, phenotype association, genomic annotation, disease ontology, biomedical ontology

## Abstract

**Motivation:** In order to create controlled vocabularies for shared use in different biomedical domains, a large number of biomedical ontologies such as Disease Ontology (DO) and Human Phenotype Ontology (HPO), etc., are created in the bioinformatics community. Quantitative measures of the associations among diseases could help researchers gain a deep insight of human diseases, since similar diseases are usually caused by similar molecular origins or have similar phenotypes, which is beneficial to reveal the common attributes of diseases and improve the corresponding diagnoses and treatment plans. Some previous are proposed to measure the disease similarity using a particular biomedical ontology during the past few years, but for a newly discovered disease or a disease with few related genetic information in Disease Ontology (i.e., a disease with less disease-gene associations), these previous approaches usually ignores the joint computation of disease similarity by integrating gene and phenotype associations.

**Results:** In this paper we propose a novel method called GPSim to effectively deduce the semantic similarity of diseases. In particular, GPSim calculates the similarity by jointly utilizing gene, disease and phenotype associations extracted from multiple biomedical ontologies and databases. We also explore the phenotypic factors such as the depth of HPO terms and the number of phenotypic associations that affect the evaluation performance. A final experimental evaluation is carried out to evaluate the performance of GPSim and shows its advantages over previous approaches.

## Introduction

The emergence of massive biomedical data offers a marvelous opportunity for the life science research and modern disease diagnosis. The wealth of knowledge contained in biomedical big data also brings great challenges, since many biologists chronically construct their biomedical database applications by using their own terms to represent biomedical knowledge. In order to create controlled vocabularies for the shared use of knowledge, a large number of biomedical ontologies such as Disease Ontology [DO (Schriml et al., 2012; Kibbe et al., 2014)] and Human Phenotype Ontology [HPO (Köhler et al., 2014)], etc., are created in the bioinformatics community. Biomedical ontologies (Lee et al., 2008; Köhler et al., 2009; Meehan et al., 2013; Groza et al., 2015; Patel et al., 2015; Denny et al., 2018; Lovering et al., 2018) reduce the complexity of life science's concepts and make innovative contributions to advance the understanding of human diseases with controllable terminology. Currently, these ontologies have been used in a variety of biomedical applications. For example, HPO-based analysis tools have been used to assist in clinical diagnosis (Westbury et al., 2015) and exon sequencing research (Peng et al., 2018), etc. In addition, by using DO, researchers build the chain knowledge base of etiology (Harrow et al., 2017; Kozaki et al., 2017) and annotate human genes to improve the coverage of disease genes' annotations (Osborne et al., 2009).

Exploring the associations (Landrum et al., 2014) among diseases by using biomedical ontologies has attracted a significant attention in biomedical domains (Zhao and Halang, 2006; Zhang et al., 2008; Zeng et al., 2017). Quantitative measures of these associations among diseases could help researchers gain a deep insight of human diseases, since similar diseases are usually caused by similar molecular origins or have similar phenotypes. Deducing the semantic similarity of disease is beneficial to reveal the common attributes (e.g., the classification of diseases, disease-related genes, disease-related symptoms, etc.) of these diseases, which could facilitate the understanding of underlying causes and improve the disease diagnoses and treatment plans. For example, the gene “SH2D3C” is one of the common genes of “Amnesia” and “Alzheimer's disease,” which reveals that they may involve the same biological processes. The greater similarity means that the more closely related these two concepts are, and that the more common information they have (Liu and Yan, 2016; Liu and Zhang, 2017; Liu et al., 2017). A good quantitative method for computing the similarity among diseases could directly help researchers obtain the information of diseases having close relationships from massive biomedical data and do the corresponding experiments for the further analysis, which could significantly reduce the experimental cost and improve the efficiency of discovering potential pathogenic mechanism and drugs.

DO regulates the controlled vocabularies about diseases, and integrates the diseases' terms and medical data through external links. It provides the accurate, non-duplicative terms with high disease coverage and has been used to compute the degree of correlation among diseases during last decade (i.e., the disease similarity) (Osborne et al., 2009). DO is usually selected as the source of disease terms for the disease similarity calculation. Several previous approaches, including those based on information content (IC) (Resnik, 1995; Lin, 1998; Schlicker et al., 2006; Wang et al., 2007; Bandyopadhyay and Mallick, 2014), ontology Directed Acyclic Graph (DAG) structure (Kim et al., 1993; Zhang et al., 2010; Santos et al., 2012) and biological function process (Mathur and Dinakarpandian, 2012; Cheng et al., 2014; Jeong and Chen, 2015; Zou et al., 2016; Yang et al., 2017; Ni et al., 2018), have been proposed with the aim to measure the disease similarity by using DO. For the IC-based approaches, Resnik (1995) use IC of the most informative common ancestor (MICA) to measure the similarity of two diseases. To improve the efficiency of the Resnik's method, Lin (1998) propose the ratio of the amount of IC of MICA and that of two DO terms and then Schlicker et al. (2006) improve the Lin's approach through the Bernoulli probability distribution to reduce the impact of shallow annotations (Li et al., 2010). However, IC-based approaches only focus on the semantic information of two terms in different layers of ontology DAG. They ignore the information from the ontology DAG structure, and it is difficult to reveal the semantic differences between two terms under the same MICA. DAG-based approaches are susceptible to shallow annotations since the shallow concepts are too generalized to have much information (Li et al., 2010). For the DAG-based approaches, Kim et al. (1993) consider that the reciprocal of the shortest distance of two disease in DAG to measure their similarity. Zhang et al. (2010) take into account not only the shortest distance, but also the depth of the least common ancestor. For the methods based on biological functional processes, BOG (Mathur and Dinakarpandian, 2012) calculates by the overlapping of related gene sets as the disease similarity. PSB (Cheng et al., 2014) takes account of the gene similarity additionally to improve BOG's performance. By adding associations obtained from external databases, BOG and PSB perform better performance than previous IC-based and DAG-based approaches. Nevertheless, they ignore the joint computation of disease similarities by integrating gene and phenotype associations, and have poor performance when evaluating disease similarities for the disease with less genetic information such as viral infectious disease (Common Wart, DOID:11165) and vein disease (Esophageal Varix, DOID:112) in DO.

To effectively evaluate the similarities of newly discovered diseases or diseases with few genetic information in current medical research (i.e., diseases with less disease-gene associations), we propose a novel semantic similarity measure method called GPSim in this paper. GPSim takes genes, diseases and phenotypes into account, and calculates the similarities by jointly utilizing their associations extracted from multiple biomedical ontologies and databases. Besides, we explore the phenotypic factors influencing the performance of GPSim. The experimental results show that, in comparison with previous similarity evaluation methods, our proposed approach has the best performance in terms of ROC (receiver operating characteristic curve) and AUC (area under curve).

## Methods

In this section, we introduce the details of our proposed method GPSim. GPSim relies on the associations of disease-gene and disease-phenotype. We firstly integrate the association data extracted from HPO, DO, and other biomedical databases, and then compute the corresponding disease similarity.

### Disease-Genetic and Disease-Phenotypic Relationship Integrations

Disease-phenotype and disease-gene-phenotype mapping relations mainly come from the HPO mapping file (Download from http://compbio.charite.de/jenkins/job/hpo.annotations.monthly/lastStableBuild/artifact/annotation/ALL_SOURCES_ALL_FREQUENCIES_diseases_to_genes_to_phenotypes.txt). The disease information is extracted from the DO, which has totally 11191 disease terms and 2,140 of them have disease-gene and disease-phenotype mapping relations, and 808 have disease-phenotype mapping relations. Additionally, we integrate the proven disease-gene relationships in the SIDD (Cheng et al., 2014) and the Dancer databases (Download from http://wodaklab.org/dancer/ downloads). Through the completely matching names and synonyms of DO terms, we identify the DO terms and obtain the corresponding disease-gene mapping from Dancer. In this scenario, the number of terms having disease-gene and disease-phenotype mapping relations is 2505.

**Figure 1:**
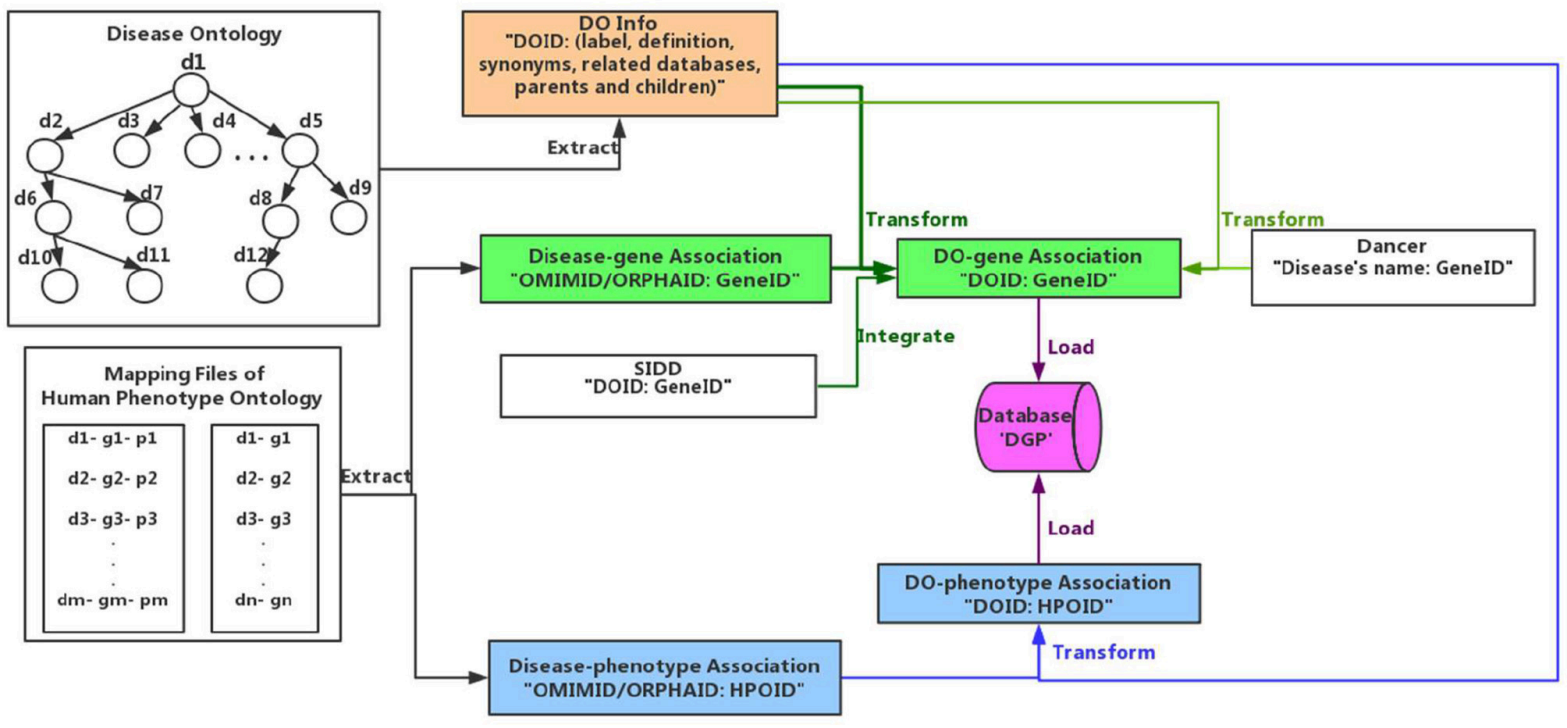
Associated data integrations.

As shown in Figure 1, we extract the disease terms including disease's id, label, definition, synonyms, related databases, parents, and children from DO (Peng et al., 2013). Then we gain the disease-gene associations from Dancer, SIDD and HPO, and their relationships among diseases and phenotypes from HPO. The format of data from Dancer is “Disease's name: GeneID.” We identify the disease term in Dancer through totally matching the disease's name, obtaining the association such as “DOID: GeneID.” The format of data obtained from SIDD and HPO is “OMIMID/ORPHA ID/DOID: GeneID”, and we get the association through matching their ID information. Similarly, we transform the disease-phenotype associations into available formats “DOID: HPOID” through the id of OMIM, Orphanet, and DO. Finally the associated data of disease-gene and disease-phenotype are loaded and integrated in the database (depicted as DGP).

### Computing the Similarity

The similarity evaluation of any two DO terms relies on disease-gene and disease-phenotype associations. We firstly compute the similarity of disease-related gene set and the similarity of disease-related phenotype set, and then integrate them as follows:

(1) $$\begin{array}{l} simGPSim\left(d1,\;d2\right)=\beta \times simGeneSet\left(G1,G2\right)\\ \;+\left(1-\beta \right)\times simHPOSet\left(P1,P2\right) \end{array}$$

Here, *simGPSim* represents the disease similarity computed by using GPSim. For two DO terms *d1* and *d2, G1*, and *G2* represent the disease-related gene sets of *d1* and *d2*, respectively. *P1* and *P2* represent the disease-related phenotype sets of d1 and d2, respectively. simGeneSet represents the similarity between *G1* and *G2*. *simHPOSet* represents the similarity between *P1* and *P2*. β is the weight tuning the contribution of genes and phenotypes to the similarities of diseases, and the value of β depends on the quality of disease-gene and disease-phenotype associations (e.g., the association number, the depth of terms in HPO) of diseases in the tested dataset.

Computing the similarity of two gene sets relies on the gene-gene similarity network. We extract the network from the HumanNet (Lee et al., 2011). The HumanNet is a probabilistic functional gene network. Each interaction in the HumanNet is a log-likelihood score (*LLS*) which measures the probability of a true functional linkage between two genes. The functional similarity of two genes by normalizing the HumanNet (denoted as *LLSN*) are computed as follows (Cheng et al., 2014):

(2) $$LLSN\left(t1,t2\right)=\;\frac{LLS\left(t1,t2\right)-LLS_{\min}}{LLS_{\max}-LLS_{\min}}$$

(3) $$sim_{gene}\left(g1,g2\right)\;=\;\{\begin{array}{ll} 1, & g1=g2\\ LLSN\left(g1,g2\right), & g1\neq g2and\;e(g1,g2)\;\in HumanNet\\ 0 & g1\neq g2and\;e(g1,g2)\;\in HumanNet \end{array}$$

Here, *LLS*_*min*_ and *LLS*_*max*_ represent the minimum and maximum in the HumanNet, respectively. *sim*_*gene*_ represents the similarity of two genes *g1* and *g2*. If there is no linkage of two genes in HumanNet, then their similarity is 0. Thus, the similarity measurement of two gene sets is defined as follows (Cheng et al., 2014):

(4) $$\begin{array}{l} sim\;GeneSet(G1,G2)\\ \;=\frac{\sum_{gi\in G1}sim_{max}\left(gi,G2\right)\;+{\sum^{\text{​}}}_{gi^{,}}{}_{\in G2}sim_{max}(gi^{,},G1)}{n\;+\;m} \end{array}$$

(5) $$sim_{max}\left(k^{,}G\right)\;=max\{ki\in G-sim\left(k,ki\right)\}$$

**Figure 2:**
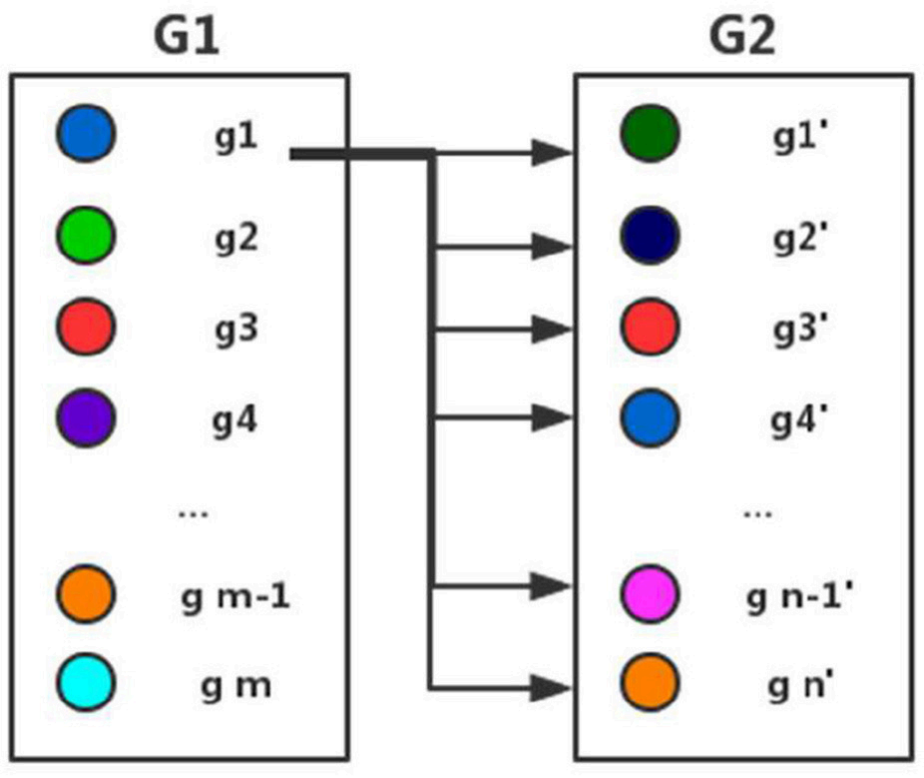
Computing the similarity between two gene sets.

Here *gi* represents a gene in the gene set *G1* and *gi'* represents a gene in the gene set *G2*. *k* represents a gene in a gene set. *G* represents a gene set and *ki* represents any gene in *G*. We define the similarity between a gene *k* and a gene set *G* as the maximum of the similarity of *k* and *ki* in *G*. As shown in Figure 2 and formula (4), we compute the similarity of every gene *gj* (*j* = 1, 2,…,m) in gene set *G1* and that of every gene g*j'* (*j* = 1, 2,…,n) and gene set *G2* respectively, and then calculate the average value of all similarities representing the similarities of two gene sets *G1* and *G2*.

Computing the similarity of two disease-related phenotype sets relies on the association of diseases and phenotypes. We could measure the similarity of two phenotype sets by their overlaps, the similarity between two phenotype sets could be defined as follows:

(6) $$\begin{array}{r} simHPOSet\left(P1,P2\right)\;=\;\frac{2\times |P1\cap P2|}{\left|P1|+|P2\right|} \end{array}$$

**Figure 3:**
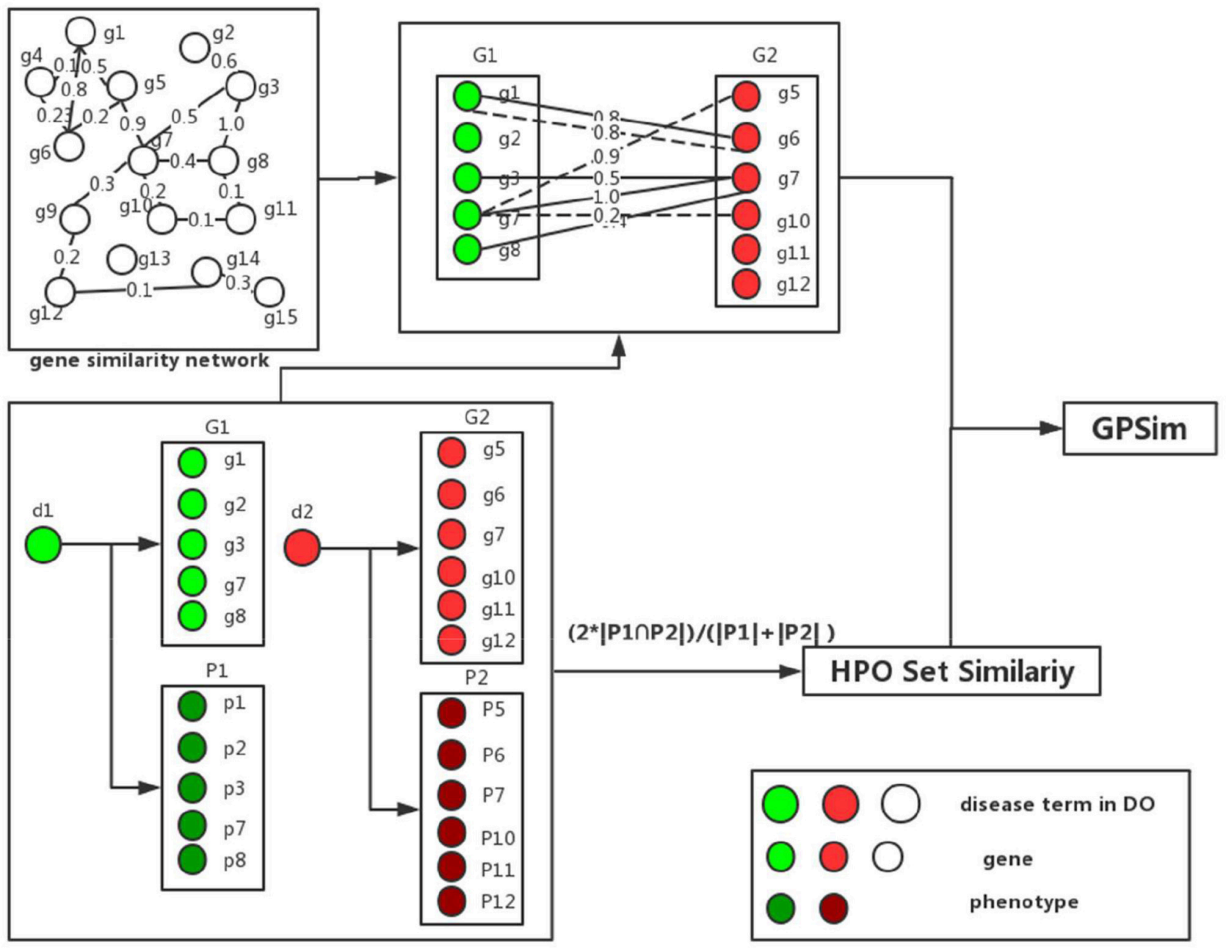
The similarity calculation by using GPSim.

The total process of the disease similarity computation is shown in Figure 3. For instant, to calculate the similarity of two disease terms, “Alzheimer's disease” (DOID:10652) and “schizophrenia” (DOID:5419). We firstly get the disease-related gene sets and disease-related phenotype sets of two diseases respectively, from the integrated DGP database in Disease-Genetic and Disease-Phenotypic Relationship Integrations. By using formula (4) and (5), the similarity of two gene sets is calculated as 0.4784. The similarity result of two phenotype sets by using formula (6) is 0.1111. Finally, we integrate the similarity of gene sets and phenotype sets by using formula (1), in this scenario the corresponding similarity value is 0.4417.

Let *N* be the total number of diseases in DO, and *K* and *L* be the sizes of disease-related gene and disease-related phenotype sets, respectively. There are *N*^2^ pairs of diseases and it costs *O*(*N*^2^) to compute all the similarities. For each disease pair, we need to compute both the similarities based on disease-related gene and disease-related phenotype sets. Calculating two diseases' similarity based on disease-related gene sets costs *O*(*K*^2^) to obtain the corresponding similarity. The intersection between two disease-related phenotype sets takes *O*(*L*). As a result, it takes *O*(*N*^2^^*^(*K*^2^ +*L*)) to compute the similarity of all disease pairs.

## Results

In our experiments, we explore the phenotypic factors including the depth of HPO terms and the number of disease-phenotype associations when each disease has few disease-gene associations and compare GPSim with previous disease similarity measurement methods, including Resnik (Resnik, 1995), Zhang (Zhang et al., 2010), BOG (Mathur and Dinakarpandian, 2012) and SemFunSim(Cheng et al., 2014).

All the experiments are performed on 2.50 GHz Intel Core i7 CPU with 8.00 GB RAM running on Windows 10 64-bit system. We implemented all the approaches in Java with JDK 1.8.0 and Python 3.0.

To provide a fair comparison with previous approaches, we select the disease pairs with disease-gene and disease-phenotype associations from the SIDD benchmark and carry out the experiments by using the tested method used in previous approach (Cheng et al., 2014). In particular, we take the disease pairs in the benchmark set as the positive examples, and randomly generate 500 disease pairs as the negative examples, combining the positive examples and the negative examples as a tested set. To reduce test error we generate 100 tested sets to compare the performance of different methods and get the average value of 100 test results. For each tested set, we calculate the similarity of each disease pair by using the Resnik's method, Zhang's, BOG, SemFunSim and GPSim, and the performance comparisons are performed by using a receiver operating characteristic curve (ROC). ROC curve is a curve drawn with true positive rate (TPR) as Y axis and false positive rate (FPR) as X axis according to a series of different dichotomies (boundary values or decision thresholds). Generally, the closer to the upper left corner the ROC is, the more accurate the corresponding method is. For showing the performance of different methods more directly, the area under curve (AUC) of the ROC was also given. The greater AUC is, the better the performance is.

**Figure 4:**
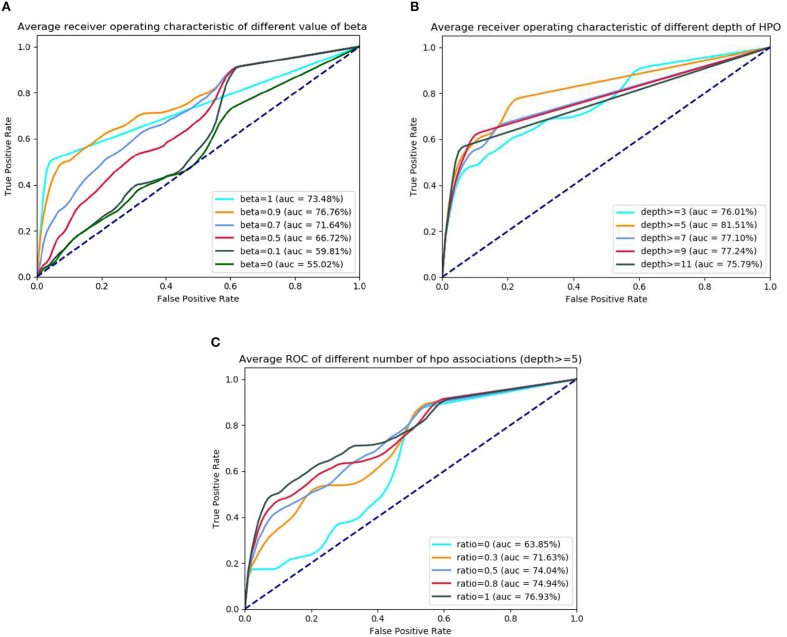
The ROC and AUC using different phenotypic factors. **(A)** ROC of different beta. **(B)** ROC of different HPO's depth. **(C)** ROC of different HPO's quantity.

In the first sets of experiments, for diseases having less genetic associations (e.g., <9) in DO, we firstly calculate the similarities by using GPSim with different values of the beta (see formula 1 in Computing the Similarity). From the results observed from Figure 4A, we see that beta value of 0.9 is an optimum threshold in the tested dataset, which also reveals that jointly using disease-gene and disease-phenotype associations could improve the effect of disease similarity measurements. In this scenario, we also investigate the impact of the phenotypic factors such as the depth of HPO terms and the number of the disease-phenotype associations. To test the impact of similarity evaluation using different depth of HPO terms, we vary the HPO terms' depth from 3, 5, 7, 9, and 11. As shown in the Figure 4B, we see that, when the HPO terms' depth is >5, after obtained the corresponding disease-phenotype associations (depth ≥5), GPSim obtains the best the performance (the AUC is 81.51%), which illustrates that the performance of calculating disease similarity is declined by using the shallow HPO disease-phenotype associations. Figure 4C shows the experimental results using different number of disease-phenotype associations in the deep layer of the HPO (e.g., depth ≥5). From the figure, we see that, the more the number of disease-phenotype associations, the better the effect of GPSim.

**Figure 5:**
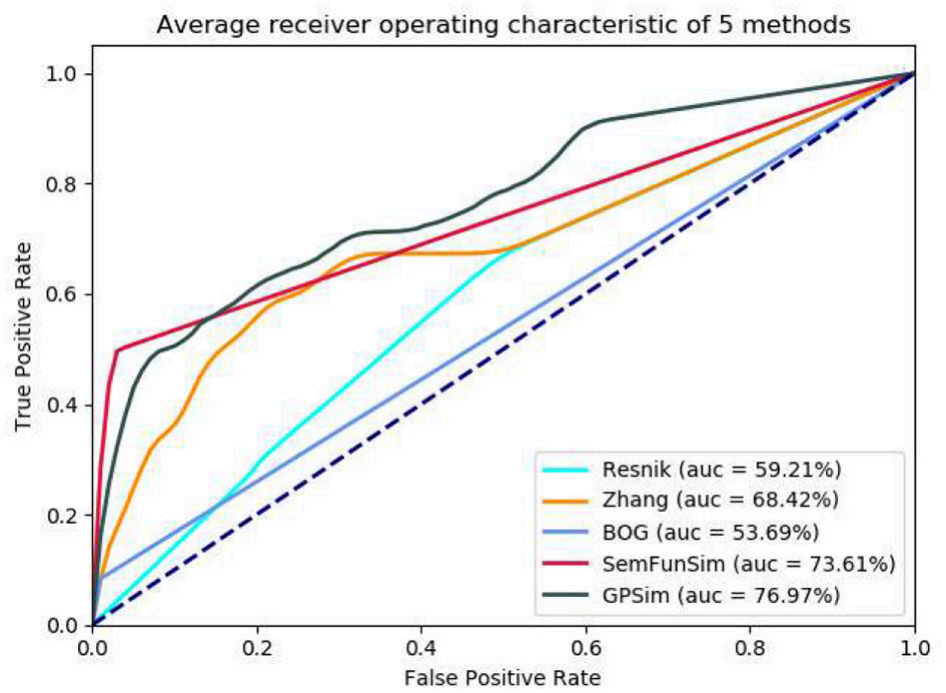
Performance comparisons based on the dataset with few genetic information.

**Figure 6:**
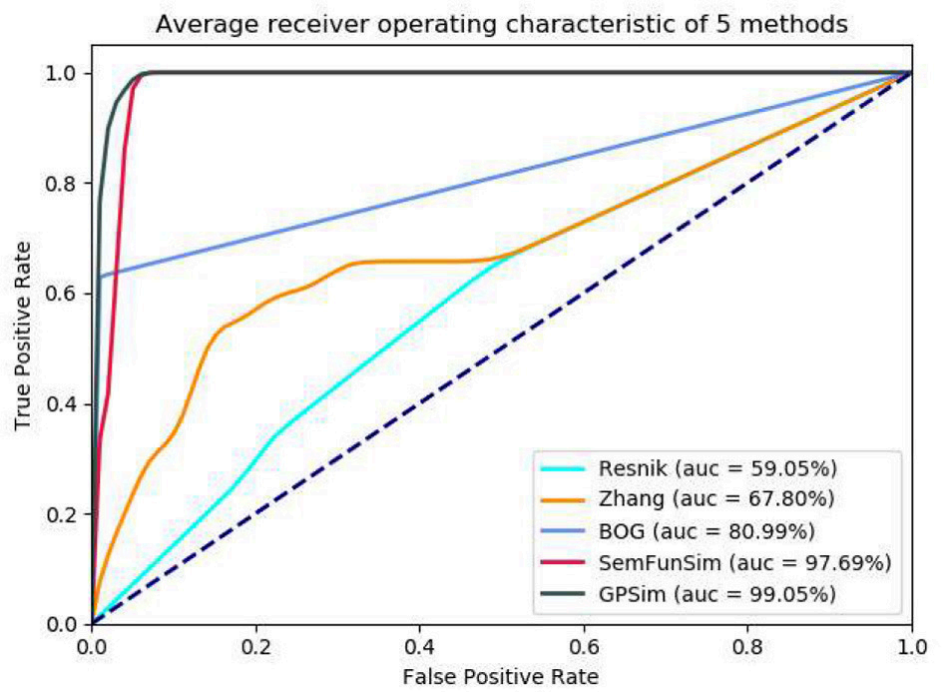
Performance comparisons based on all associations.

In the second sets of experiments, we firstly compare the performance of Resnik, Zhang, BOG, SemFunSim and GPSim in terms of ROC and the AUC, for the scenarios of diseases with few disease-gene associations (e.g., <9). As shown in Figure 5, GPSim also presents the best performance. Figure 6 shows the performance for the scenarios of diseases with multiple disease-gene associations, and the consistent results are obtained and GPSim has the best performance. In particular, we see that the AUCs of GPSim, SemFunSim, BOG, Zhang and Resnik are 99.05, 97.69, 80.99, 67.80, and 59.05% respectively. Note that, since the negative samples are randomly generated, the average values of AUC of these methods may have a 2% float, and their corresponding floating directions are consistent. This is because (i) the similarity evaluation of Resnik's and Zhang's methods are centered on Disease Ontology only, and additional information such as associations among genes and diseases are not taken into account, (ii) BOG and SemFunSim improve the similarity measurement method by adding associations among genes and diseases to alleviate information insufficiency, (iii) GPSim further integrate gene, disease and phenotype associations extracted from multiple biomedical ontologies and databases, and it jointly utilizes these associations to effectively deduce the semantic similarity. Therefore, GPSim is more suitable for the similarity evaluation, which is what we have expected.

## Conclusion

The vast amount of biomedical data has brought huge benefits to disease diagnosis and life science research, but it has also brought challenges to the understanding and searching of biological information in different disease terms. Thus, a large number of biomedical ontologies with controlled vocabularies are created for the biomedical knowledge share. Currently, quantitative measures of the associations among diseases by using biomedical ontologies have become the research hotspot. In this paper, we focus on the joint computation of disease similarities by integrating gene and phenotype associations. In particular, we propose an effective method to measure the similarity of diseases in Disease Ontology with disease-related gene and phenotype associations extracted from HPO and other biomedical databases, which calculates the similarities by jointly utilizing their associations. The final experiments show that, our proposed method has the best performance in terms of ROC and AUC, compared with previous methods. In the future, we plan to apply GPSim to the disease annotation applications for providing researchers with a more powerful annotation tool based on biomedical ontologies. Additionally, we would like to involve more information, such as gene sequence, expression information, to improve our disease similarity model.

## Data Availability

All datasets analyzed for this study are included in the manuscript and the supplementary files. The source code of GPSim is freely available at https://github.com/lyotvincent/GPSim.

## Author Contributions

JL conceived the project, conceptualized the method, designed the studies, and contributed to writing the manuscript. JL, SS, and LZ implemented the algorithms, performed the analysis and contributed to writing the manuscript. All authors read and approved the final version of the manuscript.

### Conflict of Interest Statement

The authors declare that the research was conducted in the absence of any commercial or financial relationships that could be construed as a potential conflict of interest.
